# A novel method for quantifying the rate of embryogenesis uncovers considerable genetic variation for the duration of embryonic development in *Drosophila melanogaster*

**DOI:** 10.1186/s12862-016-0776-z

**Published:** 2016-10-07

**Authors:** Barbara Horváth, Andrea J. Betancourt, Alex T. Kalinka

**Affiliations:** 1 Institut für Populationsgenetik, Veterinärmedizinische Universität Wien, Veterinärplatz 1, A-1210 Vienna, Austria; 2 Vienna Graduate School of Population Genetics, Veterinärmedizinische Universität Wien, Veterinärplatz 1, Vienna, A-1210 Austria

**Keywords:** Rate of development, Embryogenesis, *Drosophila*, DGRP, Genetic variation, GWAS

## Abstract

**Background:**

Embryogenesis is a highly conserved, canalized process, and variation in the duration of embryogenesis (DOE), i.e., time from egg lay to hatching, has a potentially profound effect on the outcome of within- and between-species competition. There is both intra- and inter-specific variation in this trait, which may provide important fuel for evolutionary processes, particularly adaptation. However, while genetic variation underlying simpler morphological traits, or with large phenotypic effects is well described in the literature, less is known about the underlying genetics of traits, such as DOE, partly due to a lack of tools with which to study them.

**Results:**

Here, we establish a novel microscope-based assay to survey genetic variation for the duration of embryogenesis (DOE). First, to establish the potential importance of DOE in competitive fitness, we performed a set of experiments where we experimentally manipulated the time until hatching, and show that short hatching times result in priority effect in the form of improved larval competitive ability. We then use our assay to measure DOE for 43 strains from the *Drosophila* Genetic Reference Panel (DGRP). Our assay greatly simplifies the measurement of DOE, making it possible to precisely quantify this trait for 59,295 individual embryos (mean ± S.D. of 1103 ± 293 per DGRP strain, and 1002 ± 203 per control). We find extensive genetic variation in DOE, with a 15 % difference in rate between the slowest and fastest strains measured, and 89 % of phenotypic variation due to DGRP strain. Using sequence information from the DGRP, we perform a genome-wide association study, which suggests that some well-known developmental genes affect the speed of embryonic development.

**Conclusions:**

We showed that the duration of embryogenesis (DOE) can be efficiently and precisely measured in *Drosophila*, and that the DGRP strains show remarkable variation in DOE. A genome-wide analysis suggests that some well-known developmental genes are potentially associated with DOE. Further functional assays, or transcriptomic analysis of embryos from the DGRP, can validate the role of our candidates in early developmental processes.

**Electronic supplementary material:**

The online version of this article (doi:10.1186/s12862-016-0776-z) contains supplementary material, which is available to authorized users.

## Background

The diversity of traits directly connected to an organism’s life cycle– life-history traits– is endless, and so are the adaptive strategies that organisms can use to increase their fitness. Because adaptation depends on the genetic variation available for selection, the genetics of life-history traits has long been a major concern of evolutionary biology, and subject to serious controversy and debate. Advances in theoretical [1, 2] and empirical quantitative genetics [3–7] have changed our view of life-history traits, enabling more realistic conclusions about the components of phenotypic variation and the forces maintaining this variation. The low heritability of complex quantitative traits is not necessarily the consequence of depleted additive genetic variation, as it had been thought previously [8–10], but often the result of extensive non-additive and environmental variance ([2, 11–13]; for a review see [14]). While many studies have contributed to our understanding of fitness and its phenotypic trade-offs, gaps in our knowledge about the complex network of fitness characters remain.

One life-history trait that has received much attention is development time (DT) in holometabolous, ectotherm organisms, such as *Drosophila* and other insects [15–24]. DT is one of the best-studied and documented life-history traits, particularly regarding the effects of environmental cues, such as temperature [15–17, 23] and crowding [18–20]. Genetic studies showed that mutations in certain genes can also influence DT [21]. Much of the interest in this trait is because it is believed to be closely related to fitness, as has been shown for *Drosophila* [9, 20, 25], particularly when larval competition is high [20]. Furthermore, because *Drosophila* is a genetic model system, it provides an especially good opportunity for understanding the genetic basis of developmental rate, with developmental control genes and their downstream counterparts being well-documented [26], as are those affecting developmental rate when disrupted [21]. Mutations resulting in hormone signaling defects in *Drosophila* [27–29], for example, can affect the larval-pupal transition, delaying or disrupting normal development. Moreover, *Drosophila* is also a useful model for understanding natural variation in developmental rate, with selection experiments revealing considerable genetic variation for DT in *Drosophila* [30–32].

Here, we are interested in the initial developmental stage of insects, taking place from the fertilization of an egg until hatching of the larva, or the duration of embryogenesis (DOE). DOE has often been treated as a fixed, species-specific developmental event, and variation in the rate of embryonic development has been considered as a passive, unavoidable consequence of abiotic factors such as temperature. In spite of that, there is a great variety of hatching patterns, and embryos as a responsive, evolving stage in the life-cycle have gained attention recently [33]. There is significant between- [17, 34, 35] and within-species variation in this trait [36, 37]. Importantly, DOE varies greatly among insects, ranging from months (e.g., 4.5 months postdiapause for *Aulocara* at 15 °C) [38] to weeks (2 weeks for *Grillus* at 29 °C) [39], days (3 days at 25 °C, *Heliconius* [40]; 7 days at 25 °C, *Parasteatoda* [41]) or hours (~22 h at 25 °C for *Drosophila* [26]).

As for DT generally, *Drosophila* is a particularly good model for understanding genetic variation in DOE, as embryogenesis itself has been the subject of intense study [17, 26, 30, 35, 36]. But, while egg-to-adult and larval development have both been well-characterized [15, 16, 18–24], variation in DOE has been relatively neglected, in part because it is not an easy trait to measure. Previous studies quantified DOE using a technique in which eggshells of the hatching larvae were counted manually [30, 35]. As this method is very labor-intensive and prone to observational errors, it also limits work to only one or two strains/populations at a time, and experimenters have only one chance to record hatching. Given these limitations, a more reliable and repeatable method adaptable to high-throughput is clearly needed to investigate this trait, and has been developed in the present study. That is, to determine whether there is within-species genetic variation for DOE in *Drosophila*, and to precisely quantify it, we established a new microscope-based phenotypic assay and measured the trait in 43 DGRP strains. We found extensive genetic variation for DOE among the strains, and showed that the majority of the identified polymorphisms retard the time to hatching.

## Methods

### Establishing the ecological importance of embryonic development time

To establish the importance of DOE for larval competition, we experimentally manipulated differences between strains in DOE by modifying the age of the eggs in one strain (RAL555: DGRP line 555) while keeping the age fixed in the other (white-eye reference strain, w^1118^). We chose this DGRP line because previous experiments showed that it is similar to the reference strain with respect to egg-to-adult viability (EAV) and DT (data not shown). We tested the competitive advantage of a head-start in DOE under both high density (HD; 175 reference/175 DGRP eggs; 350 eggs in total on 8 ml standard media) and low density (LD; 50/50 eggs; 100 eggs in total). We gave the DGRP strain different levels of advantage in DOE by collecting eggs from this strain earlier than those of the control strain. We tested three levels of advantage: (a) no advantage (“0 h”), (b) 4 h of advantage (“4 h”) and (c) 7 h of advantage (“7 h”). We assessed the fitness of the two competing strains by measuring EAV and DT under these conditions, as both traits are closely related to overall fitness [9, 20, 42]. An increase in EAV and/or decrease in DT in the strain with the advantage given, or the opposite effects for the reference strain, would confirm that DOE contributes to overall fitness in *D. melanogaster*.

### *D. melanogaster* fly strains for DOE measurements

The phenotypic response of 43 DGRP strains (*D. melanogaster* Genetic Reference Panel) [43] was measured, including all 40 strains of the initially sequenced and phenotyped core-set. These inbred *Drosophila* strains allowed us to phenotype a large number of individuals with the same genetic background, making it possible to measure a precise and robust trait mean for each strain. All flies were reared on standard molasses/soy-corn flour/agar media containing vials under low density conditions, in a single 25 °C incubator at 70–80 % relative humidity with a 12/12 h light/dark cycle.

### Experimental populations and egg collection

To measure DOE, we first age-synchronized the parental population. To do this, we set up large egg-laying population cages at 25 °C (100 mm, one cage/DGRP strain), and collected eggs on 100 mm Petri dishes filled with blackcurrant juice and agar media, sprinkled with fresh yeast. Eggs were collected over a 12 to 24 h period, and set up in bottles on standard *Drosophila* media at low density (10–15 flies/ml food). For measuring DOE, 1 to 2 day-old flies were transferred to population cages and were acclimatized at 25 °C for 2 days prior to the experiments. On days when measurements were taken, synchronized embryos were collected for an hour (between 6 and 7 pm) on blackcurrant juice plates. Two 1-h long pre-lays preceded this egg collection in order to encourage females to lay eggs they may have incubated for varying time periods [30]. Eggs were carefully washed off the plates into egg collection chambers, and transferred with a fine brush onto a 24-well cell-culture plate (see below) for imaging. Each well of the plate can accommodate ~100 embryos, however, numbers varied depending on the number of embryos collected. Each round of phenotyping was conducted with randomly chosen blocks of strains, with 2–4 of them assayed simultaneously on each array. We also phenotyped a reference strain (w^1118^, *D. melanogaster*) in parallel with the DGRP strains. The w^1118^ strain acted as a control to account for experimental noise.

### Imaging plate and imaging

The transfer and subsequent imaging with a stereoscope camera provides far more accurate and repeatable measures than those obtained manually. It reduces experimental and observational error, as well as increases the throughput of the experiment, since in this way more individuals can be measured simultaneously. Five randomly distributed wells were used for each DGRP strain, and four for the reference strain. We imaged embryos on a 24-well cell culture plate in which all wells were previously filled with 1 ml 2.5 % blackcurrant agar. Using a precisely measured amount of agar in each well facilitates subsequent imaging, because one focus setting can be used throughout the experiment, and the blackcurrant juice provides a contrasting background for the images of eggs.

Sequences of images were taken at sixteen predefined time points throughout the day, between 10 am and 8 pm (Additional file 1: Table S1). Imaging started at 10 am, equivalent to 15.5 h of DT (taking the midpoint of the 1-h long egg collection as starting point). A strong distinction exists between non-retained and retained embryos, with hatching times following a bimodal distribution [44]. Embryos that hatched before 10 am are presumably from eggs that began embryonic development while still retained in the female, and therefore were discarded from the analysis [30]. Excluding these embryos from the analysis allowed us to avoid confounding effects due to heterogeneous egg retention behavior of the strains.

Scoring was done with image analysis software (Fiji, [45]) by counting empty egg shells of the hatched larvae on consecutive images (Additional file 2: Figure S1). First, a time-lapse image stack was created from the individual pictures of each batch of eggs, then this image stack was aligned using the eggs themselves as landmarks. For counting the hatched eggs, we used the “cell-counter” plugin (http://rsbweb.nih.gov/ij/plugins/cell-counter.html).

We partitioned hatched embryos into 17 developmental categories (Additional file 1: Table S1). After excluding early-hatching embryos (<15.5 h) from the analysis, and combining late-hatching ones (>25.5 h) with the last measured time point (25.5 h), we had measurements of DOE for every strain and every replicate separately. We calculated weighted mean DOE by multiplying the number of eggs hatched at a given time point with the corresponding DOE. We calculated relative DOE by subtracting the mean reference value from the strain means. Negative values represent faster DOE compared to the reference strain, while positive values mean slower development.

### Quantitative genetic analysis

All statistical analyses were conducted using R version 3.1.2 (R Core Team 2014). To analyze variation in DOE among DGRP strains, we performed ANOVA on the untransformed data, since the normality test did not indicate non-normal residual distribution (Shapiro-test, W = 0.98, *p* = 0.07). Specifically, we fit the following linear model: *Y*_*ij*_ = μ + L_*i*_ + ε_*ij*_, where *Y*_*ij*_ is the relative mean DOE measure for the *j* ^*th*^ replicate of the *i* ^*th*^ line, L_*i*_ is the effect of line *i* (*i* = *1–43*), ε_*ij*_ is the error term for the *i* ^*th*^ line and the *j* ^*th*^ replicate and *j* is the replicate (*j = 1–3)*. The σ_L_^2^ provides an estimate of genetic variance (σ_G_^2^), and the error (ε) provides an estimate of the environmental variance (σ_E_^2^). Phenotypic variance (σ_P_^2^) is calculated according to the formula σ_P_^2^ = σ_G_^2^+ σ_E_^2^, and broad-sense heritability (*H* ^2^) was estimated as *H* ^2^ = σ_G_^2^/ σ_P_^2^. The phenotypic and genetic coefficients of variation were calculated using raw data, as CV_P_ = 100*√ (σ_P_^2^/*X*) and CV_G_ = 100*√ [(σ_P_^2^*H ^2^)/*X*], where *X* and σ_P_^2^ are the mean and variance of raw DOE estimates across all strains. To perform post-hoc analysis of the data, we used the Tukey-Kramer test implemented in the *HSD.test* test in R (package “agricolae”).

To assess the ecological importance of DOE, we first fitted the maximal, four-way ANOVA model on the Box-Cox transformed DT. The Box-Cox transformation was used to satisfy the normality and homogeneity of variance assumptions for the residuals of the fitted model, and lambda was calculated with the *powerTransform* function (package “car”). The initial, full model included four main effects [a line effect, L (*i* = 1–2); density treatment, D (*j* = 2); sex, S (*k* = 2) and the hours of advantage given to the DGRP strain, T (*l* = 3)], all of their interaction terms, and an error term (ε). After sequentially dropping non-significant terms, including several interaction terms and one main effect (sex), we obtained the simplified model below, which was used for subsequent analysis: *Y*_*ijkl*_ = μ + L_*i*_ + D_*j*_ + T_*k*_ + L_*i*_D_*j*_ + D_*j*_T_*k*_ + ε_*ijkl*_, where *Y*_*ijkl*_ is the transformed DT (Table 2A). As an independent confirmation for our model reduction, we used the *step* function in R, which uses AIC as the model selection criteria, and which produced the same reduced model as above. We also analyzed the data separately by density and advantage given to the DGRP strain (Additional file 3: Table S2).

To analyze the EAV data, we performed the same steps as for DT, but using the arcsine square-root transformed EAV ratios. In this case, however, sex was not included in the full model. The minimal adequate model obtained for EAV was *Y*_*ijk*_ = μ + L_*i*_ + D_*j*_ + L_*i*_D_*j*_ + ε_*ijk*_ (Table 2B). The linear models for DT and EAV fit the transformed data well, as indicated by the non-significant Shapiro-test results performed on the model residuals (DT: Shapiro-test, W = 0.99, *p* = 0.84; EAV: Shapiro-test, W = 0.97, *p* = 0.38).

We used correlations to gain insight into the underlying basis for the genetic variation.

If the variation is maintained due to antagonistic pleiotropy—with genes affecting one trait favorably and another trait negatively—we expect negative correlations between fitness components. If it is the result of deleterious mutations exposed by inbreeding, we instead expect positive genetic correlations between fitness components [12]. To examine such scenarios, we used a Spearman rank test for quantifying correlations between DOE and other DGRP phenotypes.

### Genome wide association study

We tested for associations between SNPs and the relative DOE measures (median of the three replicates of the relative mean DOE). Single trait measures were uploaded in February 2016 to the DGRP webtool (http://dgrp2.gnets.ncsu.edu) [43], which performed the analysis between the trait measures and the corresponding DGRP strain sequence variants. Briefly, we tested SNPs and indel markers for an effect on the *Wolbachia*—and major inversion polymorphism–adjusted single trait measures, restricting the analysis to markers with a minor allele count ≥ 4 and a per-strain coverage between 2 and 30. For every marker, we fit the model *Y*_*ij*_ *= μ + M*_*i*_ *+ ε*_*ij*_, where *M* was the effect of the polymorphic marker (SNP or indel). Multiple regression and partial least squares were used to correct for biased allelic effect estimates.

We performed calculations to assess the power of detecting large effect alleles at the level of *p* < 10^−5^ influencing DOE, following the procedure of Ivanov et al. [46]. For this, we used the *pwr.t2n.test* function in R (package “pwr”), which needs minor (*n*_*1*_) and major allele counts (*n*_*2*_) as well as a Cohen’s effect size estimate (*d*). *d* is calculated by dividing the mean phenotypic difference between the two genotypes (μ_1_–μ_2_) by the standard deviation, σ_p_, which is the square root of the sample variance, calculated by √[(*n*_*1*_-1)σ^2^_1_ + (*n*_*2*_-1)σ^2^_2_ /(*n*_*1*_ + *n*_*2*_-2)] [46].

We also performed GO analysis with GOWINDA [47] to test for any enrichment in functional gene groups with SNPs affecting DOE. With a relaxed *p*-value of 10^−4^ we included 268 variants as candidates in the analysis, for which we used the program’s gene mode, included SNPs 2000 bp up/downstream of a gene, used minimum gene number of 5 and 1,000,000 simulations. To compare rates of molecular evolution of candidate DOE genes relative to others in the *Drosophila* genome, we used estimates of *d*_*N*_, *d*_*S*_, and *d*_*N*_/*d*_*S*_ (ω) along the lineage leading to *D. melanogaster* from FlyBase (ftp://ftp.flybase.net/genomes/12_species_analysis/clark_eisen/paml/).

## Results

### Ecological importance of embryonic development time

**Fig. 1:**
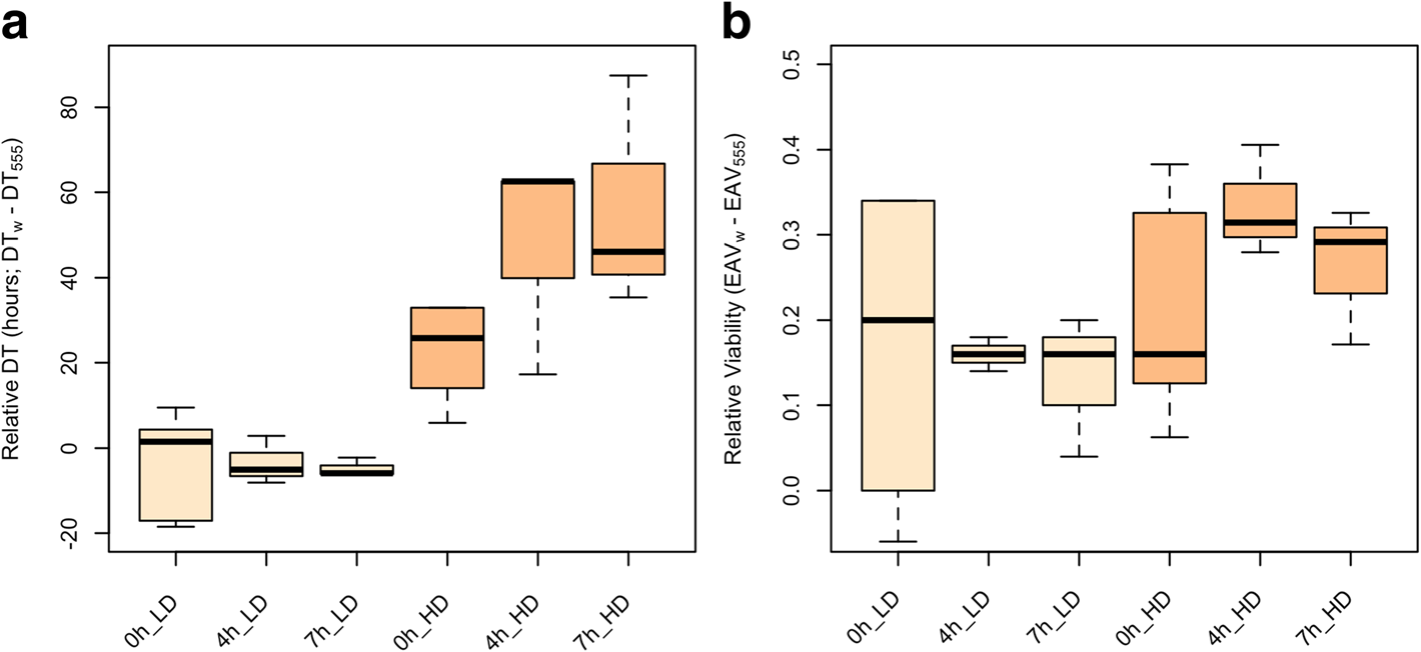
Relative DT **a** and viability **b** values for two competing genotypes measured at low (LD) and high (HD) densities. The two competing genotypes are w = reference strain, w^1118^ and 555 = DGRP line RAL555. We tested the competitive advantage of a head start in embryonic DT by giving varying levels of advantages to the DGRP strain. Fitness effects of three levels of advantage were tested: no advantage (“0 h”), 4 h advantage (“4 h”), and 7 h advantage (“7 h”). Six replicates were combined for the “0 h” data, while 3–3 replicates gave the “4 h” and “7 h” measurements

To assess the fitness consequences of shortened/prolonged embryogenesis, we manipulated the DOE for one of the strains, and measured DT and EAV for two competing genotypes under two larval densities (Table 1). Prior to data analysis, we corrected for the given advantages to the DGRP line in DOE (4 and 7 h), and subtracted the corresponding values from the overall DT of the experimentally delayed reference strain. The experiments revealed significant effects of density and genotype, as well as significant interactions between density and genotype and density and embryo age (Table 2). We found no significant effect of sex on either of the measured traits. The strain effect for DT was much smaller than that for EAV, measured as the fraction of phenotypic variance explained by DGRP line. This appears to be mainly due to the strong effect of density on variation in DT; when restricting the analysis to single density treatments, the strain effects were more similar between the two traits (Additional file 3: Table S2; phenotypic variance explained by strain for DT, low density: 56.8 %, DT, high density: 83.1 %; EAV, low density: 90.8 %, EAV high density: 91.6 %). To have a simple estimate of the fitness effect of embryo age, we calculated relative DT and EAV values by taking the difference between the two competing strains (DT_w_–DT_555_; EAV_w_–EAV_555_; Fig. 1). Higher relative DT values imply faster DT in the DGRP strain, while a smaller difference between the EAV of the two strains indicates increased viability for the DGRP strain (since viability of the reference strain was higher in all comparisons). Under benign, low-density conditions, DT was marginally faster in the reference strain, regardless of the advantage given to the DGRP strain [mean relative DT ± S.D. (in hours): −3.12 ± 11.74 (“0 h”); −3.43 ± 5.68 (“4 h”); −4.81 ± 2.25 (“7 h”); F_1,42_ = 4.300, *p* = 0.0443; Fig. 1, Additional file 3: Table S2A.1], but the embryo age did not appear to have a significant effect. However, under competitive, high-density conditions, ANOVA revealed significant main effects of both modified embryo age (F_1,42_ = 6.29, *p* = 0.0041) and genotype (F_1,42_ = 38.74, *p* = 1.89E-07; Fig. 1, Additional file 3: Table S2A.2). The difference between the two strains increased gradually from “0 h” to “7 h”, in keeping with the idea that DOE *per se* has an impact on fitness, measured as DT (Additional file 3: Table S2A.3–5). That is, increasing the advantage to the DGRP strain from “0 h” to “4 h” increased the DT difference from 34.07 ± 33.9 h (“0 h”) to 47.45 ± 26.16 h (“4 h”) (ANOVA, F_1,20_ = 3.13, *p* = 0.092). With 7 h of desynchronization, this gap became even more pronounced, the relative DT difference between the two strains increased to 56.27 ± 27.45 h (ANOVA, F_1,20_ = 12.72, *p* = 0.0019).

**Table 1:** Measurement means (±se) of development times (DT) and viabilities (EAV) for two competing genotypes

			Developmental Time (DT), in hours				Viability (EAV)	
Density	Adv	Sex	*n*_*555*_	*n*_*w*_	RAL555	w	RAL555	w
*Low*	0 h	F	93	115	634.38 ± 8.4	628.68 ± 5.04	0.567 ± 0.07	0.737 ± 0.036
	0 h	M	77	106	637.86 ± 9.66	624.84 ± 8.76		
	4 h	F	43	68	623.34 ± 7.5	630.24 ± 6	0.58 ± 0.031	0.74 ± 0.031
	4 h	M	44	43	635.34 ± 6.3	632.1 ± 8.4		
	7 h	F	49	55	627.36 ± 5.64	629.4 ± 9.36	0.6 ± 0.061	0.733 ± 0.027
	7 h	M	41	55	624.42 ± 12.36	630.72 ± 6.3		
*High*	0 h	F	256	333	742.5 ± 16.86	836.58 ± 37.68	0.399 ± 0.057	0.602 ± 0.052
	0 h	M	162	299	747.18 ± 15	820.32 ± 41.88		
	4 h	F	131	190	798.18 ± 14.04	936.06 ± 46.02	0.411 ± 0.016	0.745 ± 0.022
	4 h	M	85	201	775.68 ± 22.68	899.88 ± 49.5		
	7 h	F	129	201	762.36 ± 1.74	934.2 ± 33.42	0.446 ± 0.032	0.709 ± 0.023
	7 h	M	105	171	755.34 ± 13.86	898.86 ± 34.92

**Table 2:** Analysis of variance on development time (A) and viability (B)

*Source*	Df	Sum Sq	Mean Sq	*F* value	*P*	σ^2^ (%)
A - minimal adequate model for DT						
Density	1	1.32E-08	1.32E-08	932.695	1.30E-48***	94.60
Strain	1	1.21E-10	1.21E-10	8.530	0.0044**	0.87
Embryo age	2	4.10E-11	2.00E-11	1.441	0.2423	0.14
Density – Strain	1	4.97E-10	4.97E-10	35.167	5.82E-08***	3.56
Density – Embryo age	2	2.03E-10	1.01E-10	7.170	0.0013**	0.72
Residuals	88	1.24E-09	1.40E-11			0.10
B - minimal adequate model for EAV						
Strain	1	0.545	0.545	44.993	3.07E-08***	70.83
Density	1	0.186	0.186	15.364	3.06E-04***	24.18
Strain – Density	1	0.026	0.026	2.167	0.148	3.42
Residuals	44	0.533	0.012			1.57

*Abbreviations*: *df.* degree of freedom, *σ* ^*2*^ variance component

**p < 0.01; ***p < 0.001

Development time and viability of the two competing strains (RAL555: DGRP line 555, w: w^1118^). Adv is the advantage given to the DGRP strain (0, 4 or 7 h), *n*_*555*_ and *n*_*w*_ represents the number of individual flies measured for each genotype. Development time is calculated as the time elapsed from the day of egg collection until the emergence of the adult flies (scored daily and for males and females separately), and viability was the ratio of emerged adult flies to the initial egg numbers (50 or 175).

On the other hand, we found no evidence for a significant embryo age effect for EAV at either low or high density (ANOVA, LD: F_1,18_ = 0.033, *p* = 0.967; HD: F_1,18_ = 1.633, *p* = 0.223), and consequently no considerable increase/decrease in viability in the competing genotypes due to the given advantage/delay (Fig. 1, Additional file 3: Table S2B). Despite the fact that viability of the DGRP strain showed an increase with the increasing levels of advantage at both low and high densities (LD: 0.56–0.58–0.6; HD: 0.4–0.41–0.45), the relative viability measures showed no trend due to greater, undirected changes in viabilities of the reference strain (Tables 1 and 2).

### Genetic variation for the duration of embryogenesis in the DGRP

**Fig. 2:**
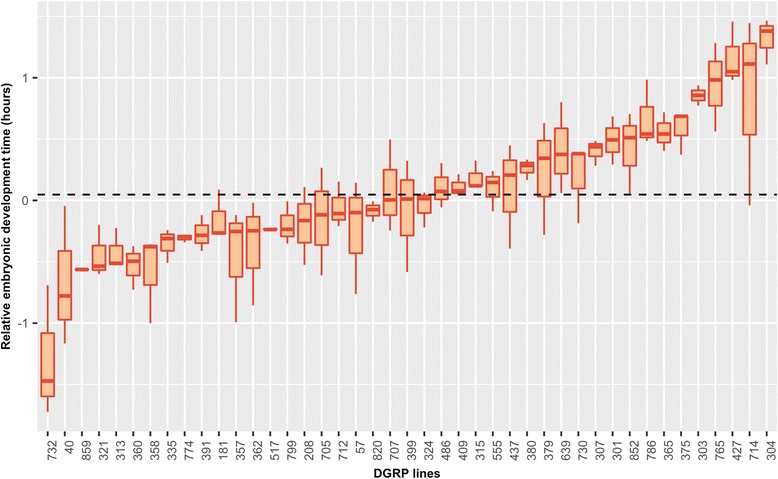
Relative embryonic development time in 43 DGRP strains. The boxplot contains relative phenotype measures in three replicates for each strain (apart from strain 303 with two replicates), compared to a reference, white-eye fly strain (w^1118^). Dashed line represents the global average across all phenotyped strains

To characterize natural genetic variation in DOE, we measured time to hatching relative to a reference (*D. melanogaster*, w^1118^ strain) for 43 DGRP strains. In all, we were able to measure DOE for 59,295 individual embryos, with 47,435 belonging to the DGRP strains and 11,860 for the reference strain. A mean ± S.D. of 1103 ± 293 embryos was measured per DGRP strain, ranging from 209 (strain 303) to 1491 (strain 321). Mean DOE across all strains was 21.69 ± 0.65 h (Additional file 4: Figure S2). The vast majority of the strains showed similarity in their DOE, with 29 of 43 DGRP strains hatching within 1 h [±0.5 h of the reference (Fig. 2, Table 3; Additional file 4: Figure S2)]. However, we found one strain (732) that was considerably faster than the reference, showing a difference of 1.471 h, and three that were up to 1.38 h slower (427, 304 and 714; Fig. 2, Table 3).

**Table 3:** Measurement means (±se) of embryonic development times for 43 DGRP strains

Strain	RAL ID	Embryonic DT	Rel. med. DOE	*n*	Egg viability
1	40	21.126 ± 0.317	−0.77839	499	0.68
2	57	21.619 ± 0.348	−0.09887	780	0.81
3	181	21.530 ± 0.116	−0.26534	871	0.92
4	208	21.683 ± 0.048	−0.16403	1215	0.97
5	301	21.973 ± 0.159	0.49353	915	0.76
6	303*	22.224 ± 0.214	0.85595	153	0.73
7	304	22.800 ± 0.069	1.37987	584	0.61
8	307	21.584 ± 0.174	0.43685	1005	0.78
9	313	21.362 ± 0.061	−0.51311	538	0.74
10	315	21.868 ± 0.045	0.11874	778	0.75
11	321	21.462 ± 0.094	−0.53585	1349	0.91
12	324	21.860 ± 0.144	0.01490	911	0.61
13	335	21.312 ± 0.24	−0.31097	1161	0.94
14	357	20.924 ± 0.366	−0.25336	1149	0.84
15	358	20.644 ± 0.015	−0.37766	520	0.92
16	360	20.690 ± 0.241	−0.49600	1183	0.91
17	362	21.503 ± 0.106	−0.24712	1095	0.95
18	365	22.412 ± 0.056	0.54123	1072	0.92
19	375	22.460 ± 0.05	0.68609	453	0.79
20	379	21.610 ± 0.412	0.34382	1200	0.88
21	380	22.050 ± 0.148	0.28426	1157	0.83
22	391	21.409 ± 0.162	−0.28503	684	0.71
23	399	21.597 ± 0.205	0.01121	1051	0.77
24	409	21.777 ± 0.356	0.07826	805	0.73
25	427	22.844 ± 0.158	1.04931	738	0.61
26	437	21.466 ± 0.291	0.20620	1083	0.87
27	486	21.329 ± 0.211	0.07350	916	0.72
28	517	21.673 ± 0.197	−0.23711	1390	0.95
29	555	21.478 ± 0.462	0.14651	855	0.84
30	639	22.195 ± 0.191	0.37511	359	0.47
31	705	21.635 ± 0.06	−0.11702	1222	0.84
32	707	21.962 ± 0.106	0.00416	669	0.65
33	712	21.803 ± 0.127	−0.10755	866	0.66
34	714	22.697 ± 0.489	1.11208	532	0.60
35	730	21.858 ± 0.133	0.38113	965	0.84
36	732	20.488 ± 0.267	−1.47141	399	0.79
37	765	22.726 ± 0.165	0.98187	739	0.65
38	774	21.601 ± 0.191	−0.29527	1227	0.94
39	786	22.351 ± 0.113	0.54107	752	0.75
40	799	21.591 ± 0.098	−0.23586	753	0.51
41	820	21.098 ± 0.086	−0.07486	1169	0.89
42	852	21.906 ± 0.204	0.51150	417	0.40
43	859	20.659 ± 0.199	−0.56327	1126	0.92

Embryonic development time for 43 DGRP strains. *n* indicates sample size (= number of embryos measured individually from egg laying until emergence of the first instar larva; non-viable and retain eggs are not included). Embryonic DT is the obtained raw duration of embryogenesis measure, while Rel. med. DOE is the relative measure (standardized with the reference strain). Egg viability was calculated as the ratio of hatched eggs and the total number of eggs transferred to the imaging plate. Non-viable eggs contain both unfertilized eggs and embryos that failed in their embryonic development. Values resulted in from three independent replicates, and in one case only two replicates (*)

We found significant differences between strains in their DOE (ANOVA, F_1,42_ = 8.311, *p* = 1.29E-16), with a high broad-sense heritability, H^2^, of 0.89. The genetic coefficient of variance (CV_G_) was 2.782 while the phenotypic coefficient of variance (CV_P_) was 2.975. The between-strain variation that underlies variation in DOE appears to be potentially complex; there were 19 different trait means among the 43 lines according to a post-hoc Tukey-Kramer test.

### GWAS

**Fig. 3:**
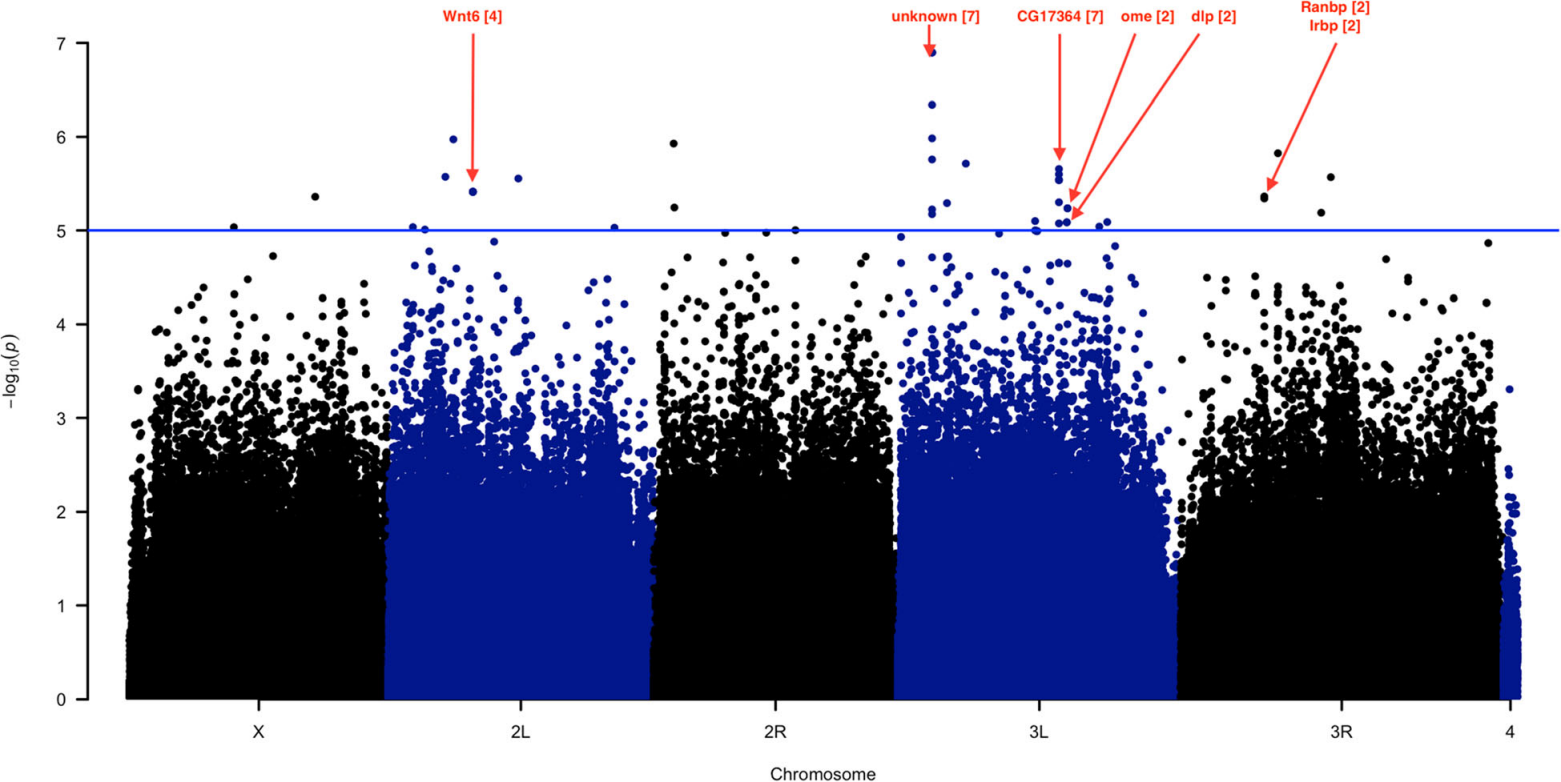
GWAS *p*-value distribution. –log_10_(*p*-value) plotted along each chromosomal position for all SNPs. Colors and letters indicate chromosome arms. Genes with multiple hits (number in brackets) as well as the highly significant peak on the chromosome arm 3 L are marked

Using the publicly available webtool (http://dgrp2.gnets.ncsu.edu) [43], we conducted a GWAS analysis on 1,526,387 genetic variants found among the 43 strains, for which the minor allele was represented in four or more strains, using single-locus analyses. At *P* < 10^−5^, we found 46 variants (45 SNPs + an indel) associated with the rate of embryogenesis (Fig. 3). The small number of strains makes our study underpowered to draw many conclusions about the genetic architecture of this trait. However, our precise and robust phenotype measures based on hundreds of individual embryos per strain can identify genes and regions putatively affecting DOE and provide a basis for subsequent follow-up studies [48].

To assess whether there is enough power to detect alleles with a large phenotypic effect on DOE, we performed power calculations, similar to Ivanov et al. [46]. We used mean phenotype values and variances of the two genotypes from our highest ranked SNP (SNP: 3L_2,989,334 (7/43), *p* = 1.26 × 10^−7^; Additional file 5: Figure S4, first panel; μ_1_ = 0.89, σ^2^_1_ = 0.138, μ_2_ = −0.086, σ^2^_2_ = 0.18). Despite using only 43 strains, we showed that we had some power to detect variants with large effects, depending on the frequency of the minor allele (MAF) –which ranged from 9 to 43 % for the 46 candidate SNPs uncovered in this analysis. For example, using a *P*-value cut-off of 10^−5^, we had 31 % power to detect a variant with an effect size equal to 1 h of difference in DOE and a MAF of 10 %. For alleles with higher MAFs, we had more power: we had 86 % power to detect variants of this effect size with a MAF of 20 %, up to 98 % power with a MAF of 40 % (Additional file 6: Figure S5).

SNPs significantly associated with variation in DOE are presented in Additional file 7: Table S3. Half of the significant SNPs (25 of 46) occurred in close proximity to each other, and are likely non-independent (or in linkage disequilibrium), resulting in a total of 27 candidate genes (Fig. 3; Additional file 7: Table S3). Among these, 11 have no known function, though the biological functions of 16 genes have been described previously. Most of the annotated polymorphisms (52 %) are intronic; if causal, the effect of these intronic SNPs may be due to their effects on gene expression, as introns can harbor regulatory elements [49].

We also examined the data for a relationship between DOE and the presence of the intracellular bacteria *Wolbachia* and common large chromosomal inversions, both of which occur in these lines. We found no effect of *Wolbachia* infection status on the rate of embryonic DT (ANOVA, F_1,2_ = 3.35, *p* > 0.05; Additional file 8: Figure S3). The DGRP lines have been typed for several chromosomal inversions [50]: among the 16 inversions that have been identified in the DGRP [50], 11 are monomorphic in our sample of 43 lines. The remaining 5 inversions (In.2 L.t, In.2R.NS, In.3R.P, In.3R.K and In.3R.Mo) show some extent of polymorphism, and two of the 5 were associated with DOE (In.2R.NS, ANOVA, F_1,3_ = 3.434, *p* = 0.04; In.3R.K, F_1,3_ = 12.39, *p* = 0.001; Additional file 9: Figure S6). We evaluated if these inversions correspond to the genomic location of any candidate genes. Two genes, *Mur89F* (in In.3R.K) and *Sema*-*2b* (in In.2R.NS) occur within the significant inversions, and in both cases the identified variant is prevalent in the sample (MAF = 46.5 % and MAF = 27.5 %, respectively). However, for both inversions, very few of the lines carry the inverted karyotypes– only 4 strains harbor In.2R.NS in either homozygous or heterozygous form, and only one line carries In.3R.K. Thus, the significant association between DOE and inversion karyotype should be treated with caution.

The majority of the identified candidate genes, 15 of 27 are embryonic genes, having the highest expression in early, intermediate or late embryos among all the developmental stages (Additional file 7: Table S3; data from [51]). Most SNPs affected DOE negatively (Additional file 5: Figure S4). However, in two cases (one variant in the *Mur89F* gene, and one associated with genes *CG7341* and *CG32195*), the derived allele was associated with a significant decrease in DOE in the lines carrying the allele (Additional file 5: Figure S4). While *Mur89F* is only expressed in late-stage embryos (after stage 13 [52]) and in later developmental stages (in larvae and pre-pupae [51]), both *CG7341* and *CG32195* are embryonic genes, with *CG7341* having the highest life-time expression in 4–6 h old embryos (Additional file 7: Table S3). GO analysis (*p* < 10^−4^: 268 variants) does not reveal any significantly enriched categories after correcting for multiple testing. However, three-quarters of the 268 mutations occurred in genes that were related to development, regulation and morphogenesis (data not shown). We also studied the molecular evolution of our candidate DOE genes identified by means of the ratio of non-synonymous (*d*_*N*_) to synonymous (*d*_*S*_) substitutions rates (ω), and compared it to other genes in the *Drosophila* genome. Consistent with purifying selection acting on these genes, embryonic genes had ω < < 1, similar to that of other protein-coding sequences in the genome (median rate for embryonic gene = 0.075, other genes = 0.063, W = 67,946, *p* = 0.675).

Among the candidate genes with defined biological functions, there was an association with a non-synonymous mutation in the *cana* gene (chr2L, pos: 11,276,472, GWAS *p* < 2.8 × 10^−6^). This embryonic gene might play a crucial role in the timing of development as it is involved in biological processes such as metaphase/anaphase transition of mitotic cell cycle and microtubule-based movements. A non-synonymous SNP was found in the *Ranbp9* gene (chr3R, pos: 7,238,756, GWAS *p* < 4.6 × 10^−6^), a gene involved in nuclear protein transport activity. Six of 43 strains carried four intronic SNPs in the w*nt6* gene, which affects processes such as neuron differentiation and cell fate commitment. An intronic SNP was found in the *hid(W)* gene in 18 of 43 strains. This gene is a well-described apoptosis activator (proapoptotic) gene. Moreover, this gene is also associated with head involution, the mechanism of the rearrangement of head segments at the anterior tip of the fly embryo [53].

Of the significant GWAS hits, eight of 46 fall into the same, 1.2 kB long region of the 3 L chromosome arm, including the five SNPs with the lowest *p*-values (chr3L, pos: 2,989,334–2,990,517). This region lacks annotation, and no known genes are in the immediate proximity of these SNPs (±5000 bp). The eight SNPs form a distinct haplotype (with the same seven DGRP lines carrying all of the mutations). To investigate the possible impact of these SNPs on gene expression, we used PROMO [54, 55] to map putative transcription factor binding sites (TFBS) in our region. We compared two haplotypes: sequence of the DGRP lines with the major alleles (in all cases, the reference *D. mel* sequence in Flybase) and the sequence of the 7 lines carrying the 8 minor alleles. The minor haplotype shows loss of two *doublesex* binding sites, and the gain of several diverse TFBSs: *deformed* (2), *tailless* (2), *paired* (1), *ftz* (1) and *dorsal* (1), proteins with crucial functions in normal development (The Interactive Fly database). Further, of the two closest flanking genes, one (*cpt2,* carnitine palmitoyltransferase 2) has its peak expression in early, 0–6 h embryos. (The downstream gene, *CG2113* has no detectable expression in embryos [51]). Though not conclusive, these results suggest a possible mechanism for the effect of these SNPs on DOE, via regulatory changes affecting *cpt2* expression.

### Correlations with other traits

**Fig. 4:**
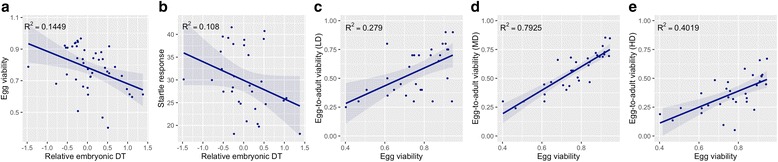
Correlations between relative embryonic DT and other measured traits in the DGRP. **a** Negative relationship between egg viability and relative embryonic DT, correlation: *R* ^*2*^ = 0.1449, *p* = 0.0068; **b** Negative correlation between embryonic DT and startle response (*R* ^*2*^ = 0.108, *p* = 0.05). Strong positive correlation between egg viability and egg-to-adult viability, measured under **c** low, **d** medium and **e** high larval densities. Correlations (C: R^2^ = 0.279, *p* = 0.001; D: R^2^ = 0.7554, *p* = 1.38E-10; E: R^2^ = 0.3851, *p* = 0.0001) are shown

To determine the relation between DOE and other known characteristics of these strains, we correlated DOE (single, median strain values) with other traits measured in the DGRP. We found a significant negative relationship between egg viability (measured as the ratio of hatched vs. total egg number in our phenotype measures) and DOE (ρ = −0.46, *p* = 0.002, Fig. 4a), implying that slow embryonic development is coupled with low egg viability. Startle response [43] is also negatively correlated with DOE (ρ = −0.39, *p* = 0.03, Fig. 4b). We found no correlation between DOE and DT [20], but we found that strains with the longest DOE are also the ones with the longest DT at both high and low densities (data not shown). However, short DOE did not result in shorter overall DT. Similarly, we tested for a relationship between EAV and egg viability measured for the same subset of lines [20]. We found a strong positive correlation between the EAV and egg viability at both low (ρ = 0.49, *p* = 0.005, Fig. 4c), medium (ρ = 0.87, *p* = 1.38E-10, Fig. 4d) and high density (ρ = 0.66, *p* = 7.55E-05, Fig. 4e). These results suggest that egg viability may be the determining factor in overall pre-adult viability in these lines.

It has been shown previously that fly species with longer DTs tend to have larger genomes [56]. As TE content is a major determinant of genome size, we hypothesized that overall TE content might be an important factor altering DOE. However, DOE did not correlate with either total TE content, or the number of novel or unique insertions [43] in these lines. We tested if there is a direct relationship between genome size [7] and DOE, but we did not find one (ρ = −0.012, *p* = 0.94), even though the subset of 43 lines was equally variable in genome size as the entire set of 211 strains (W = 4247, *p* = 0.51), providing us with some power to detect a possible correlation.

Egg retention– the ability of female flies to withhold fertilized eggs in their reproductive tracts –can have a major impact on DOE. Therefore, we also developed a way to measure this in the DGRP (in preparation), and tested whether the traits are correlated. We found no relationship between egg retention and DOE, when excluding early hatching embryos (<15.5 h) from the analysis (ρ = −0.15, *p* = 0.331). However, the relation between DOE and egg retention is significant when including early hatching embryos (ρ = −0.64, *p* = 5.01E-06). The lack of correlation between DOE and egg retention confirms that we chose an appropriate cut-off for excluding retained embryos, therefore our DOE measures are not confounded by the retention phenotype differences between strains.

## Discussion

We developed a novel assay to measure the duration of embryogenesis (DOE) and found significant genetic variation for this trait in the DGRP. Compared with the previously used, time- and labor-intensive method [30], our assay makes it possible to quantify DOE for large numbers of embryos and strains. While previous measurements were based on a small number of observations (44 – 113 per species in [35]; 81–470 per population in [30]), we were able to obtain robust phenotype measures with our method (mean ± S.D. of 1103 ± 293 per DGRP strain). Another advantage of imaging over the manual counting is that images are taken in rapid succession, minimizing noise and error in the measurements. Moreover, since the images can be stored for a long time, data can be subtracted and analyzed at a later time point, which is not possible with the manual counting.

We used our newly developed assay to study genetic variation in the DGRP for DOE, and to find possible candidate genes and proteins contributing to variation in the rate of embryogenesis. The DGRP resource [43] is valuable for studying how variation in the genome maps to phenotypic differences. The vast amount of phenotypic information available for this panel of strains, combined with genetic and transcriptomic data, makes this a powerful resource. Here, we found extensive variation in DOE in a subset of DGRP strains, translating to a 15 % difference in developmental rate between the slowest and fastest strains, comparable to the reduction of egg-to-adult DT in multigenerational selection experiments (in [57] 17 %; in [58] 24–32 %). Moreover, the variation was largely genetic in this experiment, with strain accounting for 89 % of the total phenotypic variance. This strong strain effect on DT has also been observed in lines containing *P*-element induced mutations (84 % in [21]). Such a strong strain effect is not unexpected for broad-sense heritabilites, measured on inbred lines under strictly controlled conditions.

Because complete genome sequences are available for all DGRP strains, we were able to conduct genome-wide association mapping to identify potential candidate genes that may influence the rate of *Drosophila* embryogenesis. Developmental timing is based on molecular mechanisms and molecules such as cell cycle components, cell-signaling factors and hormones [59]. Accordingly, we can find genes with previously described biological functions among our candidates that play a part in cell-cycle (*cana*), receptor activity (*Octbeta2R*, *Ranbp9*, *tho2*), cell signaling (*Wnt6*, *dlp*) and transcription (*Wnt6*, *Zpr1*). Naturally, genes with known functions in development and morphogenesis would be strong candidates for controlling DOE. Seven of the 16 genes with known functions (*Jon25Bii, cana, Wnt6, pad, hid(W), dlp* and *Lar*) are involved in developmental processes, indicating that besides their previously described roles they may be important in modulating DOE. Moreover, the available developmental transcriptome data [51] suggest that the majority of the genes, 15 of the 27 have their highest expression in embryos among all the developmental stages.

We found that the majority of significant sequence variants caused retardation in DOE. However, two alleles caused decrease in DOE (*Mur89F* intronic variant; *CG7341* intronic SNP). *Mur89F* is a mucin-related protein, known to be expressed in salivary glands of *Drosophila* embryos [52]. As many mucins are expressed in various tissues throughout embryogenesis, their potential role in organ development has been proposed [52]. While the function of the *CG7341* gene is yet to be described, its predominant embryonic expression suggests its role lying in early developmental processes. *Mur89F* is also one of the two candidate genes that can be found within segregating inversions. Many fitness traits show associations with inversion genotypes [50, 60], and in concordance with this, we found association between DOE and two inversion polymorphisms. Inversions span large regions of the genome, and can have a much higher amount of substitutions then the genome average, therefore taking into account inversions is necessary when assessing the genetic basis of traits.

Genes that are involved in highly conserved functions, such as developmental patterning and morphogenesis, are expected to be highly constrained [61]. Instead, altering the expression of crucial developmental genes through chromatin modifications or changes in transcription factor binding activity is a safer, yet effective way to control phenotypes. Consistent with this idea, most candidate SNPs, with only two exceptions, are found in non-coding gene regions (introns, up- and downstream from genes) and are potentially affecting embryogenesis via gene expression. Half of the SNPs in our sample can be found in intronic gene regions, and thus, any effect they have are likely to be mediated by gene expression [62]. Neyfakh and Hartl [30] proposed, that the plastic relationship between developmental rate and temperature suggests that variation affecting embryonic development time must be common in natural populations. In agreement with this, the average minor allele frequency in our sample was 23 % (9–46.5 %).

Our results are consistent with a general trend in the literature suggesting that rapid development, at least in *Drosophila*, is associated with high fitness. Zwaan et al. [37] found that in selection experiments, slow-developing flies showed reduced viability compared to the control and fast-developing strains. Similarly, we found a negative correlation between egg viability and relative DOE, which suggests that the relationship between high viability and rapid development also holds for embryos. Further, we mimicked the advantages of rapid embryonic development and showed that this resulted in faster overall development time when larval competition was high. The advantage experienced by a strain established early in a culture can be thought of as a kind of intraspecific priority effect [63, 64]. Priority effects describe the effect of a community initiating species on the later arriving ones, and are well known and studied in ecology literature (for a review see [63]), in particular for plant [65–67] and aquatic communities [68, 69]. While most studies focus on interspecific interactions, the importance of intraspecific effects has been described as well [68, 70]. The observed delayed/de-synchronized development is indicative of a strong competitive interaction, and can be seen as a form of intraspecific inhibitive priority effect [63, 64]. More importantly, we also found that this effect is context- and trait-dependent: the head-start caused no difference in DT of the competing lines under benign conditions (LD), but had pronounced effects when competition was high (HD). On the other hand, we observed a small but gradual increase in viability of the line with the head-start, regardless of the experimental conditions. Such context-dependency has also been shown for *Daphnia* species [70] and planktonic protists [68], and can have important implications for species relying on rapid growth, such as *Drosophila*. Priority effects increase the competitive strength of organisms and can facilitate the establishment and adaptation of otherwise less fit genotypes [70]. Finally, the strong positive correlation between egg viability and egg-to-adult viability in the studied lines suggests that embryonic viability is an important determinant of overall viability. Our results show that embryogenesis is important for fitness, and altered DOE can have consequences for other life-history traits.

## Conclusions

In this work, we studied genetic variation in a trait that we argue is ecologically relevant. For this, we developed a new, microscope- and computer-based method for quantifying genetic variation for the duration of embryogenesis (DOE) in *Drosophila melanogaster*, using the DGRP mapping panel. By characterizing DOE for more than 59,000 embryos, we found extensive variation for this trait in a subset of 43 lines. The obtained 3-h difference between the slowest and fastest strain translates to a 15 % difference in developmental rate within a single *Drosophila* species, comparable to the reduction of egg-to-adult development time in previous multigenerational selection experiments. We also show that DOE itself is ecologically important, and altered DOE can cause changes in other fitness components. With our GWAS analysis, we identified genes that may influence DOE, with the majority of these being embryonic genes and playing part in cell-cycle, receptor activity and cell signaling. We also suggest the role of being involved in developmental processes for genes with unknown biological functions. Further functional assays, or transcriptomic analysis of embryos from the DGRP can validate the role of our candidates in early developmental processes.

## Additional files

Additional file 1: Table S1. Assay of measuring the duration of embryogenesis—imaging time points and corresponding embryonic development times (PDF 51 kb)
Additional file 2: Figure S1. Time-lapse images of a sub-replicate measuring the embryonic development time of a DGRP strain (335) (PDF 719 kb)
Additional file 3: Table S2: Results of ANOVA on DT and EAV (PDF 118 kb)
Additional file 4: Figure S2. Representation of the raw phenotypic variation for embryonic development time in 43 DGRP strains (PDF 119 kb)
Additional file 5: Figure S4. Box plots of the significant SNP associations in the GWA analysis (PDF 94 kb)
Additional file 6: Figure S5. Results of the power calculations to detect associated SNPs with effect size of 1 h of difference in the duration of embryogenesis (PDF 110 kb)
Additional file 7: Table S3. Top GWAS results for embryogenesis length and the corresponding DGRP phenotypes (PDF 124 kb)
Additional file 8: Figure S3. The effects of *Wolbachia pipientis* infection on the measured phenotype (PDF 111 kb)
Additional file 9: Figure S6. Inversion polymorphisms and their effect on DOE (PDF 170 kb)

## Abbreviations

ANOVA: Analysis of variance
DGRP: *Drosophila* Genetic Reference Panel
DOE: Duration of embryogenesis
DT: Egg-to-adult development time
EAV: Egg-to-adult viability
HD: High density
LD: Low density
MAF: Minor allele frequency
