# Supplementary material for: A novel method for quantifying the rate of embryogenesis uncovers considerable genetic variation for the duration of embryonic development in Drosophila melanogaster
Source: BMC Evol Biol. 2016 Oct 7;16:200. doi: 10.1186/s12862-016-0776-z (PMC5054588; doi:10.1186/s12862-016-0776-z)
Supplement: Additional file 1: Table S1. — Assay of measuring the duration of embryogenesis—imaging time points and corresponding embryonic development times (PDF 51 kb) [file 12862_2016_776_MOESM1_ESM.pdf]

Table S1. Assay of measuring the length of embryogenesis – imaging time points and corresponding embryonic development times. Images were taken hourly from 10 am until 1 pm, then in every half an hour throughout the hatching peak, and again hourly from 6 to 8 pm. The last time point of the image stack is 10 am the following morning: embryos which hatch between 8 pm and 10 am are later added to the last development time group (25.5 h), despite their embryogenesis lasted longer than this.

Imaging time point	10:00 AM	11:00 AM	12:00 PM	1:00 PM	1:30 PM	2:00 PM	2:30 PM	3:00 PM	3:30 PM	4:00 PM	4:30 PM	5:00 PM	5:30 PM	6:00 PM	7:00 PM	8:00 PM	10:00 AM
Image number	_1	_2	_3	_4	_5	_6	_7	_8	_9	_10	_11	_12	_13	_14	_15	_16	_17
Corresponding embryonic development time	<15.5 h	16.5 h	17.5 h	18.5 h	19 h	19.5 h	20 h	20.5 h	21 h	21.5 h	22 h	22.5 h	23 h	23.5 h	24.5 h	25.5 h	> 25.5 h
