# Supplementary material for: A novel method for quantifying the rate of embryogenesis uncovers considerable genetic variation for the duration of embryonic development in Drosophila melanogaster
Source: BMC Evol Biol. 2016 Oct 7;16:200. doi: 10.1186/s12862-016-0776-z (PMC5054588; doi:10.1186/s12862-016-0776-z)

Figure S1. Time-lapse images of a sub-replicate measuring the embryonic development time of a DGRP strain (335). Six images have been split into individual pictures from an aligned image stack and used to demonstrate the method we used. The first image was taken shortly after the egg-transfer, therefore all the eggs are intact and unhatched. The following four images correspond to time point 9, 10, 11 and 12 (Table S1), as the numbers from the cell counter indicate, marking the hatching/hatched eggs in the images. The 6th, last image was taken the following morning, making it possible to quantify the number of unhatched eggs, which were used for calculating egg viability.

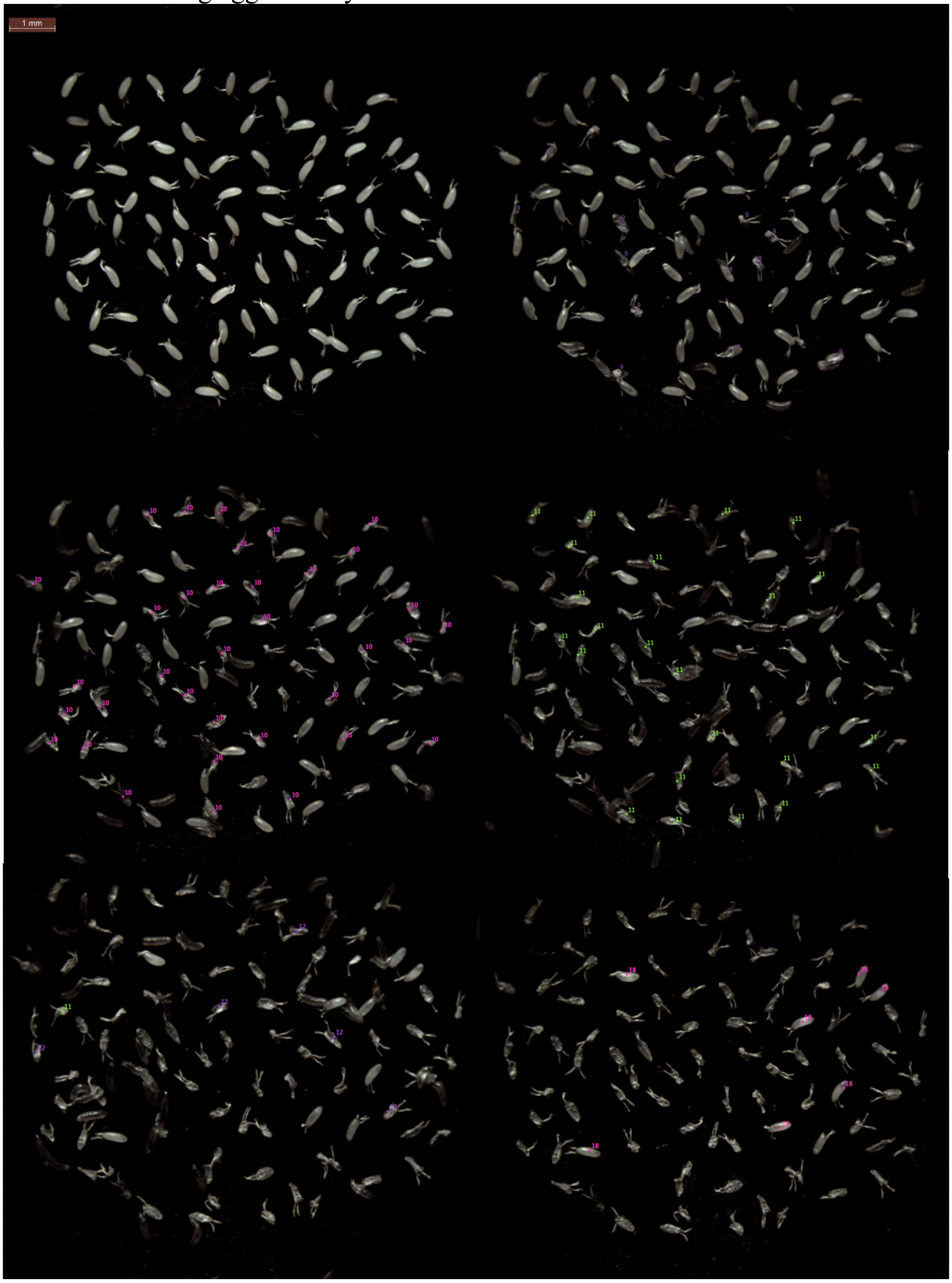

Supplement: Additional file 2: Figure S1. — Time-lapse images of a sub-replicate measuring the embryonic development time of a DGRP strain (335) (PDF 719 kb) [file 12862_2016_776_MOESM2_ESM.pdf]
