# Supplementary material for: A novel method for quantifying the rate of embryogenesis uncovers considerable genetic variation for the duration of embryonic development in Drosophila melanogaster
Source: BMC Evol Biol. 2016 Oct 7;16:200. doi: 10.1186/s12862-016-0776-z (PMC5054588; doi:10.1186/s12862-016-0776-z)
Supplement: Additional file 3: Table S2: — Results of ANOVA on DT and EAV (PDF 118 kb) [file 12862_2016_776_MOESM3_ESM.pdf]

Table S2: Results of ANOVA on DT (A) and viability (B). Prior to variance decomposition, a Box-Cox transformation was applied to the DT data (lambda values are shown), and an arcsine square root transform was applied to EAV ratios. σ^2 (%) estimates are based on Mean Sums of Squares. df.: degree of freedom; σ^2: variance component. *p < 0.05; **p < 0.01; ***p < 0.001.

Source	**Df**	**Sum Sq**	**Mean Sq**	***F* value**	***P***	**σ2 (%)**
**A – Development time (DT)**						
**A.1 – LD – lambda = -4.312378**						
RAL	1	4.75E-12	4.75E-12	4.300	*0.0443**	56.83
Embryo age	2	4.71E-12	2.36E-12	2.132	0.1312	28.18
RAL – Embryo age	2	3.00E-13	1.49E-13	0.135	0.8744	1.78
Residuals	42	4.64E-11	1.105E-12			13.21
**A.2 – HD – lambda = -3.748706**						
RAL	1	5.85E-10	5.85E-10	38.737	*1.89E-07****	83.08
Embryo age	2	1.90E-10	9.49E-11	6.287	*0.00409***	13.49
RAL – Embryo age	2	1.80E-11	9.00E-12	0.597	0.55494	1.28
Residuals	42	6.34E-10	1.51E-11			2.15
**A.3 – "0h" – lambda = -4.206**						
RAL	1	4.00E-12	4.00E-12	1.841	0.1818	0.68
Density	1	5.67E-10	5.67E-10	258.016	*5.077E-20****	95.64
RAL:Density	1	1.96E-11	1.96E-11	8.947	*0.00454***	3.31
Residuals	44	9.66E-11	2.20E-12			0.37
**A.4 – "4h" – lambda = -3.968312**						
RAL	1	1.01E-11	1.01E-11	3.128	0.09222.	0.67
Density	1	1.46E-09	1.46E-09	453.635	*3.24E-15****	96.69
RAL:Density	1	3.68E-11	3.68E-11	11.406	*0.0029***	2.43
Residuals	20	6.45E-11	3.20E-12			0.21
**A.5 – "7h" – lambda = -2.453138**						
RAL	1	5.95E-08	5.95E-08	12.720	*0.0019***	2.10
Density	1	2.60E-06	2.60E-06	555.390	*4.61E-16****	91.68
RAL:Density	1	1.72E-07	1.72E-07	36.660	*6.43E-06****	6.05
Residuals	20	9.36E-08	4.70E-09			0.17
**B – Egg-to-adult viability (EAV)**						
**B.1 - LD**						
RAL	1	0.094	0.094	11.785	*0.00297***	91.55
Age_of_egg	2	0.00053	0.00027	0.033	0.96741	0.26
RAL:Age_of_egg	2	0.00087	0.00044	0.055	0.94703	0.43
Residuals	18	0.14427	0.00801			7.76
**B.2 - HD**						
RAL	1	0.2485	0.24852	32.073	*2.26E-05****	90.84
Age_of_egg	2	0.0253	0.01265	1.633	0.223	4.62
RAL:Age_of_egg	2	0.0093	0.00465	0.600	0.56	1.70
Residuals	18	0.1395	0.00775			2.83

B.3 - "0h"

RAL	1	0.13923	0.13923	11.116	*0.00331***	56.29
Density	1	0.09378	0.09378	7.488	*0.01272**	37.91
RAL:Density	1	0.00183	0.00183	0.146	0.70615	0.74
Residuals	20	0.25049	0.01252			5.06

B.4 - "4h"

RAL	1	0.11571	0.11571	89.290	*1.29E-05****	80.03
Density	1	0.01306	0.01306	10.080	*0.0131**	9.03
RAL:Density	1	0.01452	0.01452	11.2	*0.0101**	10.04
Residuals	8	0.01037	0.0013			0.90

B.5 - "7h"

RAL	1	0.07387	0.07387	25.829	*0.000951****	73.80
Density	1	0.01538	0.01538	5.379	*0.048969**	15.37
RAL:Density	1	0.00798	0.00798	2.791	0.133367	7.97
Residuals	8	0.02288	0.00286			2.86
