# Supplementary material for: A novel method for quantifying the rate of embryogenesis uncovers considerable genetic variation for the duration of embryonic development in Drosophila melanogaster
Source: BMC Evol Biol. 2016 Oct 7;16:200. doi: 10.1186/s12862-016-0776-z (PMC5054588; doi:10.1186/s12862-016-0776-z)

Figure S2. Representation of the phenotypic variation for embryonic development time in 43 DGRP strains. The boxplot contains raw phenotypic measurements in three replicates for each strain (apart from strain 303 with two replicates), where each replicate is a combined measure of 300 individual embryos on average (min=32, max=568). Dashed line represents the global average (21.69 hours) across all phenotyped strains.

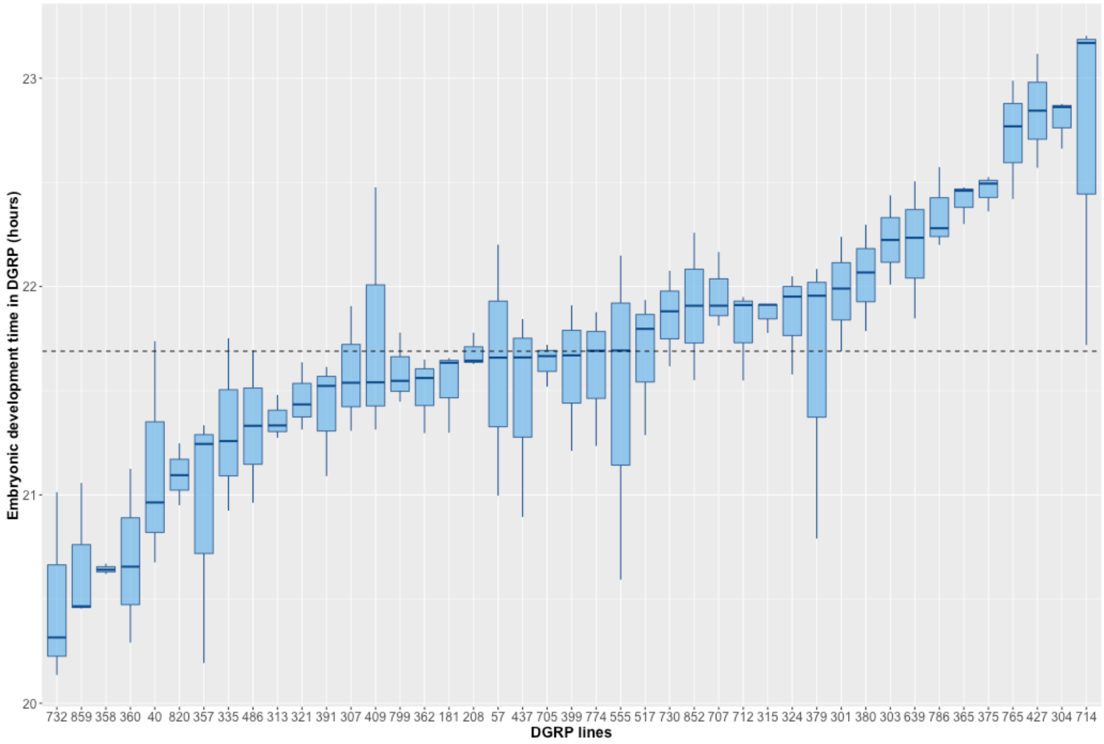

Supplement: Additional file 4: Figure S2. — Representation of the raw phenotypic variation for embryonic development time in 43 DGRP strains (PDF 119 kb) [file 12862_2016_776_MOESM4_ESM.pdf]
