# Supplementary material for: A novel method for quantifying the rate of embryogenesis uncovers considerable genetic variation for the duration of embryonic development in Drosophila melanogaster
Source: BMC Evol Biol. 2016 Oct 7;16:200. doi: 10.1186/s12862-016-0776-z (PMC5054588; doi:10.1186/s12862-016-0776-z)

Figure S4. Box plots of the significant SNP associations in the GWA analysis. In cases where multiple variants are assigned for a single gene/region the plot is based on the highest ranked associated SNP. For each SNP, the lines are partitioned into two haplotype groups (representing either the major, reference allele (0, yellow box) or the minor, derived allele (2, orange box). The *y*-axis represents the measured relative embryonic development time values (median phenotype measures for each line). The number of lines with the minor allele variant is noted in brackets following the gene name in the title of the individual plots. Box plots are ordered by significance level.

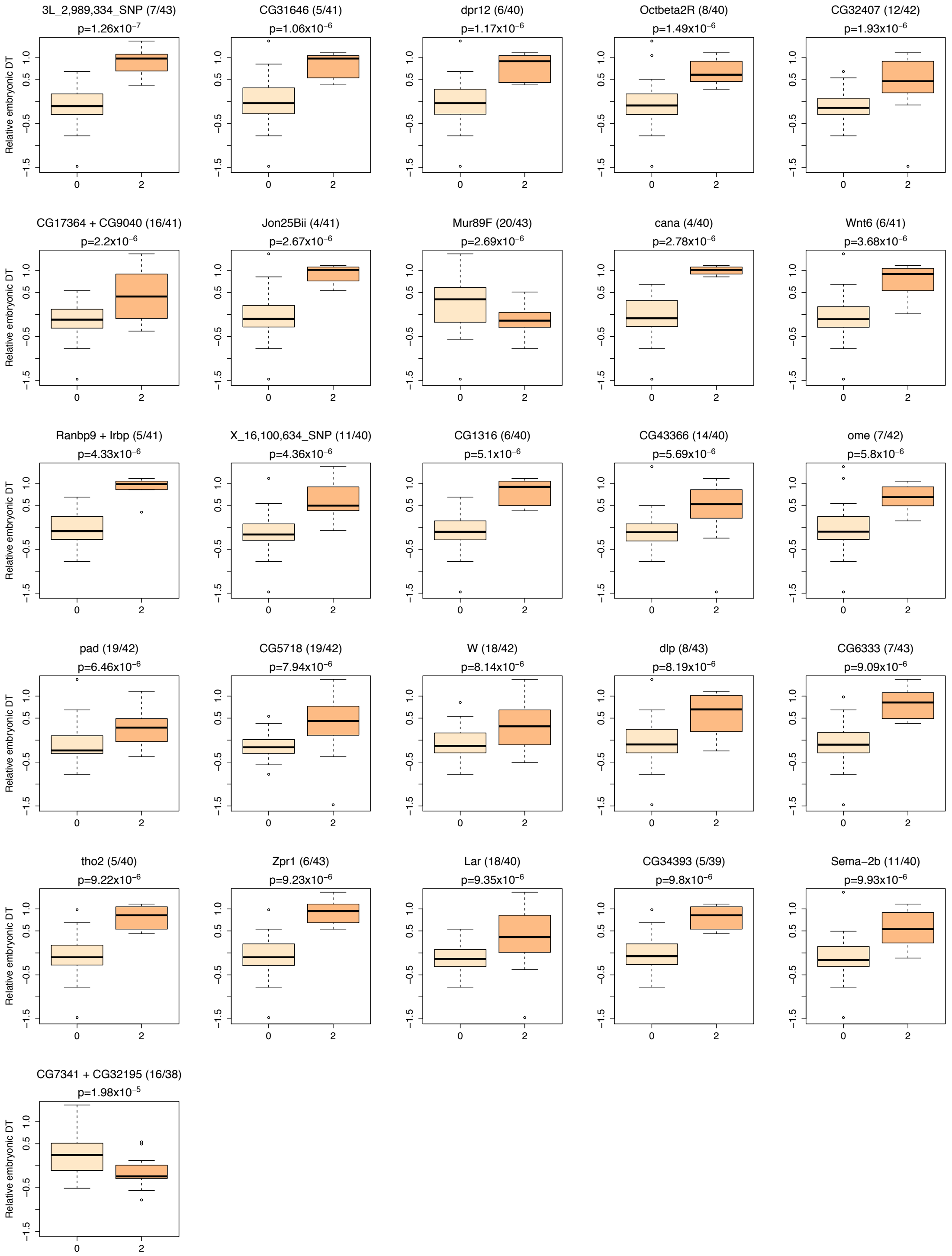

Supplement: Additional file 5: Figure S4. — Box plots of the significant SNP associations in the GWA analysis (PDF 94 kb) [file 12862_2016_776_MOESM5_ESM.pdf]
