# Supplementary material for: A novel method for quantifying the rate of embryogenesis uncovers considerable genetic variation for the duration of embryonic development in Drosophila melanogaster
Source: BMC Evol Biol. 2016 Oct 7;16:200. doi: 10.1186/s12862-016-0776-z (PMC5054588; doi:10.1186/s12862-016-0776-z)

Figure S5. Results of the power calculations to detect associated SNPs with effect size of 1 hour of difference in the length of embryogenesis (at the level of p = 10^{-5}). Cohen's d=2.335, σ_P=0.413, μ1=0.89, $\sigma^2{}_1$=0.138, μ2=-0.086, $\sigma^2{}_2$=0.18. Red vertical line shows the DGRP sample size (43).

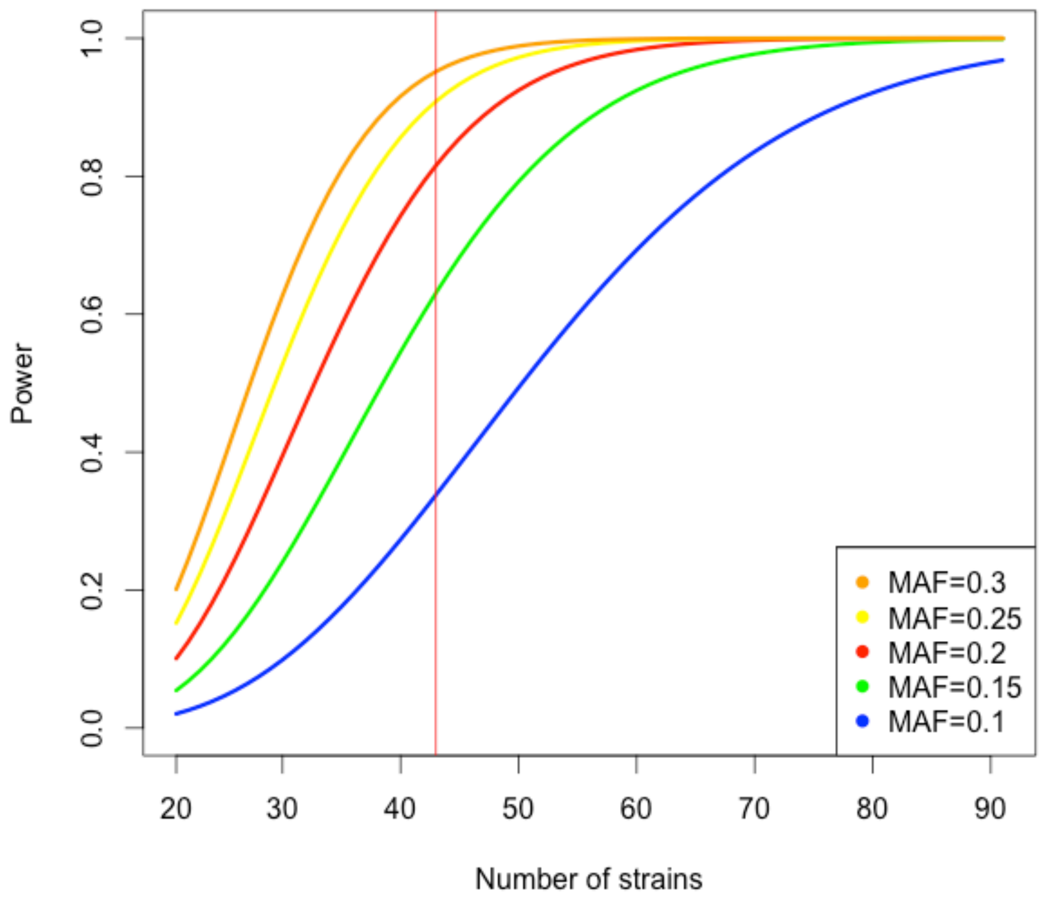

Supplement: Additional file 6: Figure S5. — Results of the power calculations to detect associated SNPs with effect size of 1 h of difference in the duration of embryogenesis (PDF 110 kb) [file 12862_2016_776_MOESM6_ESM.pdf]
