# Supplementary material for: A novel method for quantifying the rate of embryogenesis uncovers considerable genetic variation for the duration of embryonic development in Drosophila melanogaster
Source: BMC Evol Biol. 2016 Oct 7;16:200. doi: 10.1186/s12862-016-0776-z (PMC5054588; doi:10.1186/s12862-016-0776-z)
Supplement: Additional file 7: Table S3. — Top GWAS results for embryogenesis length and the corresponding DGRP phenotypes (PDF 124 kb) [file 12862_2016_776_MOESM7_ESM.pdf]

Table S3. Top GWAS results for embryogenesis length and the corresponding DGRP phenotypes. Colours indicate SNPs which affect the same genes and/or lay in the same genomic region in close proximity of each other.

Position	Minor allele	Major allele	Ref. allele	Mixed model p-val	Flybase ID	Gene ID	Genomic annotation	Function	Max expression	Embryonic expression
2989334	A	G	G	1.26E-07	NA	NA				
2990007	C	G	G	1.26E-07	NA	NA				
2990383	A	C	C	1.26E-07	NA	NA				
2990421	G	A	A	4.55E-07	NA	NA				
2990517	C	T	T	1.03E-06	NA	NA				
5647785	A	T	T	1.06E-06	FBgn0051646	DIP-theta	INTRON	unknown	Embryo 22-24 h	late
1746616	GTTCG	G	GTTCG	1.17E-06	FBgn0085414	dpr12	UTR_3_PRIME	sensory perception	Embryo 22-24 h	late
8419426	T	A	T	1.49E-06	FBgn0038063	Octbeta2R	INTRON	Receptor and TF activity	Pupae 4d	late
2989987	C	T	T	1.74E-06	NA	NA				
5916148	G	C	C	1.93E-06	FBgn0052407	CG32407	SYNONYMOUS_CODING	unknown	Embryo 12-14 h	intermed
13982689	A	C	A	2.20E-06	FBgn0036391	CG17364	INTRON	unknown	Male 30 d	late
					FBgn0036394	CG9040	DOWNSTREAM	unknown	Larva L3	late
13982684	T	G	T	2.51E-06	FBgn0036391	CG17364	INTRON	unknown	Male 30 d	late
4953245	G	A	A	2.67E-06	FBgn0031654	Jon25Bii	UPSTREAM	neurogenesis; proteolysis	Larva L3	late
13000981	T	C	T	2.69E-06	FBgn0038492	Mur89F	INTRON	chitin metabolic process	Larva L2	-
11276472	T	A	A	2.78E-06	FBgn0040233	cana	NON_SYNONYMOUS_CODING	metaphase/anaphase transition of mitotic cell cycle; microtubule-based movement	Embryo 0-2 h	early
13982667	G	A	G	2.88E-06	FBgn0036391	CG17364	INTRON	unknown	Male 30 d	late
13982671	A	AT	A	2.88E-06	FBgn0036391	CG17364	INTRON	unknown	-||-	-||-
					FBgn0036394	CG9040	DOWNSTREAM	unknown	Larva L3	late
13982673	C	T	C	2.88E-06	FBgn0036391	CG17364	INTRON	unknown	Male 30 d	late
					FBgn0036394	CG9040	DOWNSTREAM	unknown	Larva L3	late
7339060	G	A	G	3.86E-06	FBgn0031902	Wnt6	INTRON	regulation of transcription; Wnt signaling pathway; axis specification; anterior/posterior pattern specification	Embryo 6-10 h	intermed
7339108	C	A	C	3.86E-06	FBgn0031902	Wnt6	INTRON	-||-	-||-	-||-
7339117	C	T	C	3.86E-06	FBgn0031902	Wnt6	INTRON	-||-	-||-	-||-
7339135	T	C	T	3.86E-06	FBgn0031902	Wnt6	INTRON	-||-	-||-	-||-
7238785	A	C	C	4.33E-06	FBgn0037894	Ranbp9	SYNONYMOUS_CODING	intracellular protein transport	Embryo 0-4 h	early
					FBgn0011774	Irbp	DOWNSTREAM	double-strand break repair via nonhomologous end joining; telomere maintenance	Embryo 0-2 h	early
16100634	C	A	C	4.36E-06		NA	NA			
7238756	A	G	G	4.55E-06	FBgn0037894	Ranbp9	NON_SYNONYMOUS_CODING	intracellular protein transport	Embryo 0-4 h	Embryo 0-4 h
					FBgn0011774	Irbp	DOWNSTREAM	double-strand break repair via nonhomologous end joining; telomere maintenance	Embryo 0-2 h	early
13982692	T	C	T	5.00E-06	[FBgn0036391	CG17364	INTRON	unknown	Male 30 d	late
					FBgn0036394	CG9040	DOWNSTREAM	unknown	Larva L3	late
4284668	G	A	G	5.10E-06	FBgn0035526	CG1316	SYNONYMO	unknown	Embryo 4-6 h	early

							US_CODING			
1808297	T	G	T	5.69E-06	FBgn0263109	CG43366	UTR_5_PRIME	unknown	Embryo 12-14 h	intermed
14724027	A	G	G	5.80E-06	FBgn0259175	ome	INTRON	proteolysis	Embryo 16-18 h	intermed
14724028	A	G	G	5.80E-06	FBgn0259175	ome	INTRON	-||-	-||-	-||-
2990408	C	T	T	5.96E-06		NA	NA			
12169384	C	G	G	6.46E-06	FBgn0038418	pad	SYNONYMOUS_CODING	chaeta morphogenesis; negative regulation of transcription	Embryo 0-2 h	early
2990411	T	C	C	6.70E-06		NA	NA			
11917168	A	G	A	7.94E-06	FBgn0036222	SdhAL	SYNONYMOUS_CODING	respiratory electron transport; tricarboxylic acid cycle	Male 30 d	-
18166079	A	C	C	8.14E-06	FBgn0003997	W	INTRON	embryonic development; eye morphogenesis; neurogenesis; imaginal disc-derived appendage morphogenesis	Prepupae 24 h	intermed
14661465	G	A	G	8.19E-06	FBgn0041604	dlp	INTRON	positive regulation of Wnt signaling pathway; regulation of growth; neurological system process; post-embryonic organ morphogenesis; sensory organ development	Embryo 6-8 h	intermed
14661508	A	T	A	8.19E-06	FBgn0041604	dlp	INTRON	-||-	-||-	-||-
13982681	T	G	T	8.41E-06	FBgn0036391	CG17364	INTRON	unknown	Male 30 d	late
					FBgn0036394	CG9040	DOWNSTREAM	unknown	Larva L3	late
17478881	A	C	C	9.09E-06	FBgn0036731	CG6333	INTRON	unknown	Male 30 d	-
2140972	C	A	A	9.22E-06	FBgn0031390	tho2	SYNONYMOUS_CODING	oogenesis; mRNA export from nucleus	Embryo 2-4 h	early
9055188	G	A	A	9.23E-06	FBgn0030096	Zpr1	SYNONYMOUS_CODING	transcription from RNA polymerase I promoter; terminal branching; lumen formation, open tracheal system	Larva L1	early + intermed + late
19604018	T	C	C	9.35E-06	FBgn0000464	Lar	INTRON	biogenesis; cell adhesion; embryo development; regulation of nervous system development; regulation of cell shape	Prepupae 24 h	early + late
3184408	T	G	G	9.80E-06	FBgn0085422	CG34393	INTRON	unknown	Embryo 18-24 h	late
12295627	C	A	A	9.93E-06	FBgn0264273	Sema-2b	INTRON	olfactory bulb axon guidance; dendrite guidance	Prepupae 24 h	intermed
11917123	G	A	A	9.98E-06	FBgn0036222	CG5718	SYNONYMOUS_CODING	unknown	Male 30 d	-
18109582	C	G	G	1.98E-05	FBgn0036777	CG7341	INTRON	unknown	Embryo 4-6 h	early
					FBgn0052195	CG32195	DOWNSTREAM	unknown	Larva L3	early + intermed + late
