# Supplementary material for: A novel method for quantifying the rate of embryogenesis uncovers considerable genetic variation for the duration of embryonic development in Drosophila melanogaster
Source: BMC Evol Biol. 2016 Oct 7;16:200. doi: 10.1186/s12862-016-0776-z (PMC5054588; doi:10.1186/s12862-016-0776-z)

Figure S3. The effects of *Wolbachia pipientis* infection on the measured phenotype. (A) Relative mean DT measured for 43 DGRP lines. Black colour indicates no Wolbachia infection (22 lines), while blue colour is assigned for infected strains (21 lines). (B) Relative mean DT for the two *Wolbachia* groups. Black and blue are mean phenotypes among non-infected and infected lines, respectively. There is no effect of *Wolbachia* infection on the length of embryogenesis.

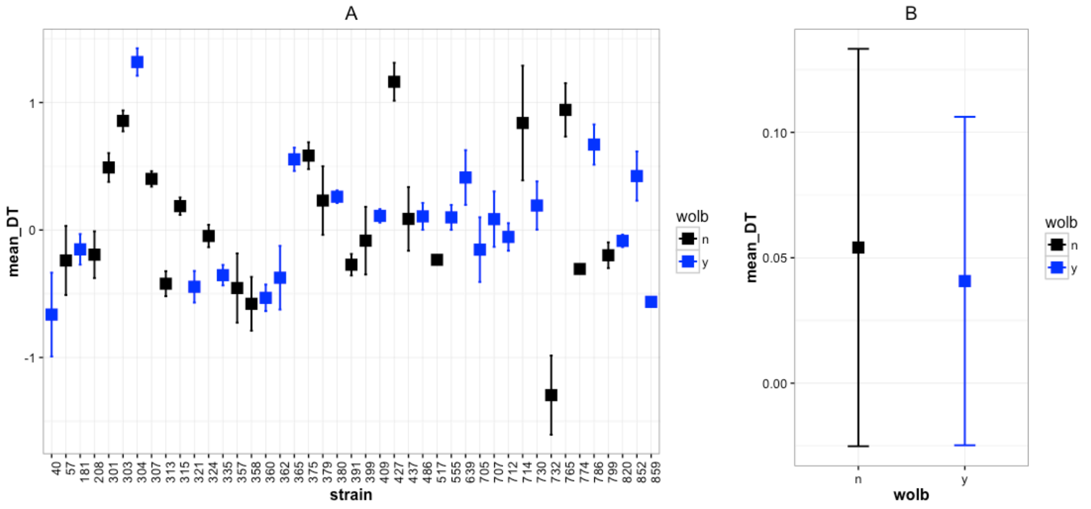

Supplement: Additional file 8: Figure S3. — The effects of Wolbachia pipientis infection on the measured phenotype (PDF 111 kb) [file 12862_2016_776_MOESM8_ESM.pdf]
