# Supplementary material for: A novel method for quantifying the rate of embryogenesis uncovers considerable genetic variation for the duration of embryonic development in Drosophila melanogaster
Source: BMC Evol Biol. 2016 Oct 7;16:200. doi: 10.1186/s12862-016-0776-z (PMC5054588; doi:10.1186/s12862-016-0776-z)

Figure S6. Segregating inversions in the 43 DGRP lines. *y* axis shows the relative median DOE measures, INV = inversion karyotype (homozygous), INV / ST = heterozygote for the inversion, ST = standard configuration in a homozygous form. Plot titles show name of the inversion, brackets indicate the number of genotypes in each group. Where the number of lines is low each RAL ID is shown individually.

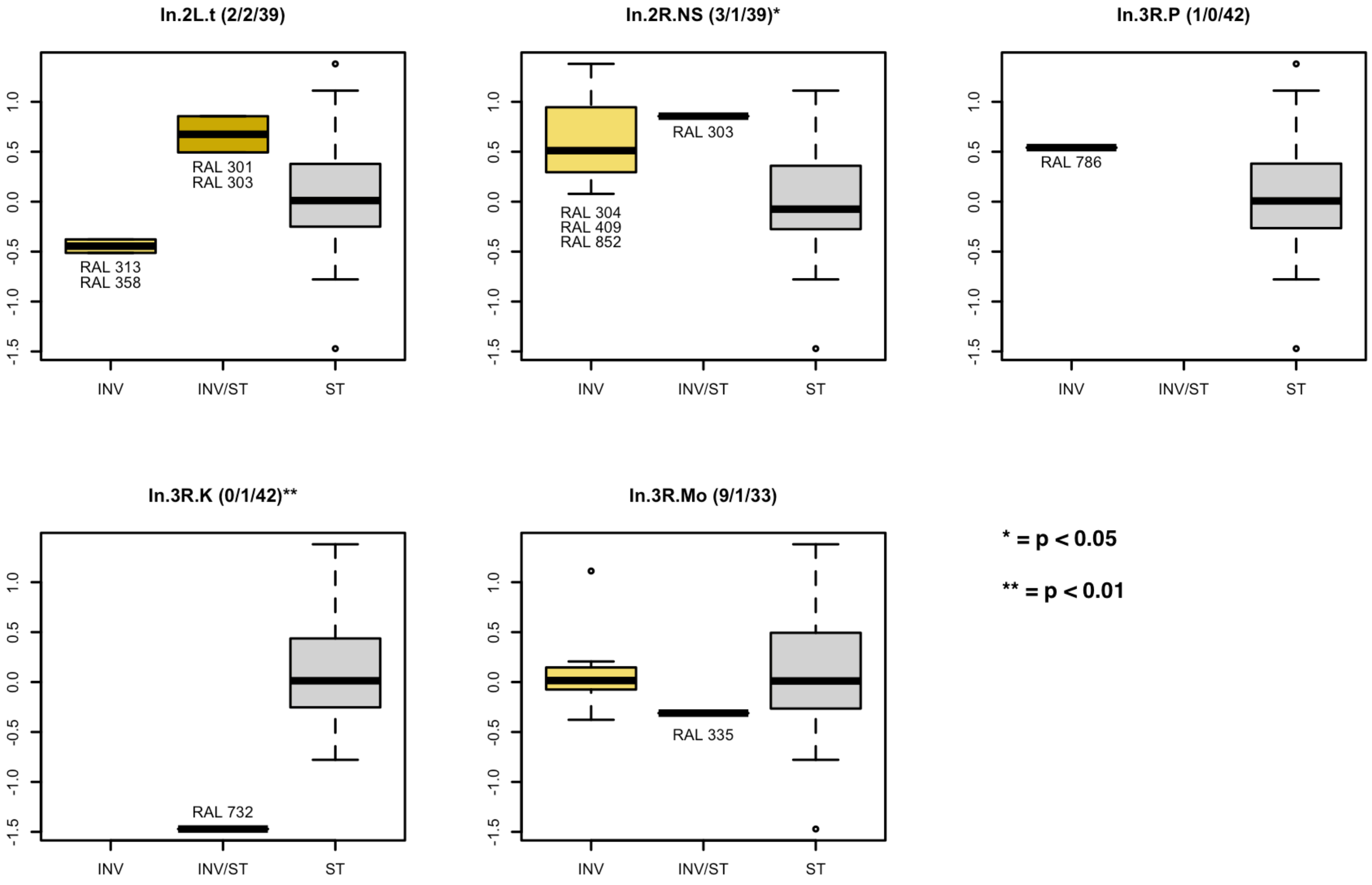

Supplement: Additional file 9: Figure S6. — Inversion polymorphisms and their effect on DOE (PDF 170 kb) [file 12862_2016_776_MOESM9_ESM.pdf]
